# MERIT: a mentor reflection instrument for identifying the personal interpretative framework

**DOI:** 10.1186/s12909-021-02579-x

**Published:** 2021-03-04

**Authors:** Lianne M. Loosveld, Pascal W. M. Van Gerven, Erik W. Driessen, Eline Vanassche, Anthony R. Artino

**Affiliations:** 1 Department of Educational Development & Research, School of Health Professions Education, Faculty of Health, Medicine and Life Sciences, Maastricht University, Universiteitssingel 60, 6229 ER Maastricht, the Netherlands; 2 Faculty of Psychology and Educational Sciences, campus Kulak, University of Leuven, Etienne Sabbelaan 51, P.O. Box 7654, 8500 Kortrijk, Belgium; 3 Department of Health, Human Function, and Rehabilitation Sciences, The George Washington University School of Medicine and Health Sciences, 2600 Virginia Avenue NW, Suite 104, Washington, DC 20037 USA

**Keywords:** Mentoring, Systematic reflection, Faculty development, Personal interpretative framework, Survey study

## Abstract

**Background:**

Essential to the professional development of mentors is making explicit and critically challenging the knowledge and beliefs underpinning their mentoring practice. This paper reports on the development of a survey instrument called MERIT, MEntor Reflection InstrumenT, which was designed to support mentors’ systematic reflection on the how, what and why of their practice.

**Methods:**

In 2019, a twenty-item survey instrument was developed and piloted. Initial validation data (*N* = 228) were collected by distributing the survey through the authors’ network. An exploratory factor analysis (EFA) was conducted and internal consistency reliability coefficients were calculated.

**Results:**

The Principal Axis EFA with Direct Oblimin rotation (Delta = 0) resulted in four factors: 1) supporting personal development, 2) modelling professional development, 3) fostering autonomy, and 4) monitoring performance. The four factors explained 43% of the total variance of item scores. The Cronbach’s alphas for the subscale scores were between .42 and .75.

**Conclusions:**

The MERIT can help mentors reflect on their beliefs and professional knowhow. These reflections can serve as input for the faculty development initiatives mentors undertake, which may ultimately improve their knowledge and skills as a mentor.

**Supplementary Information:**

The online version contains supplementary material available at 10.1186/s12909-021-02579-x.

## Background

Initiatives aimed at supporting the professionalization of mentors in higher education are growing [1–3]. This increased support of mentors’ development is encouraging as mentors have a key role in the learning and development of young health professionals, and therefore make valuable contributions to health professions education [4–8]. Building on the long tradition of research on the professional development of teachers (see, e.g., Kelchtermans [9],Vanassche and Kelchtermans [10]) we argue that initiatives designed to support mentors’ professional development should not only encourage changes in mentors’ practice, but also challenge them to interrogate their own thinking about the *how* and *why* of their practice. Without such deep reflection, and associated shifts in thinking, professional development risks becoming a simple “tips and tricks” exercise and lacks sustained impact on mentors’ practice [11]. This paper adds to this challenge by reporting on the development and initial validation of the ‘MEntor Reflection InstrumenT’ (MERIT), a survey instrument designed to make implicit knowledge and beliefs about mentoring explicit, and encourage systematic reflection on the how and why of one’s practice.

**Fig. 1:**
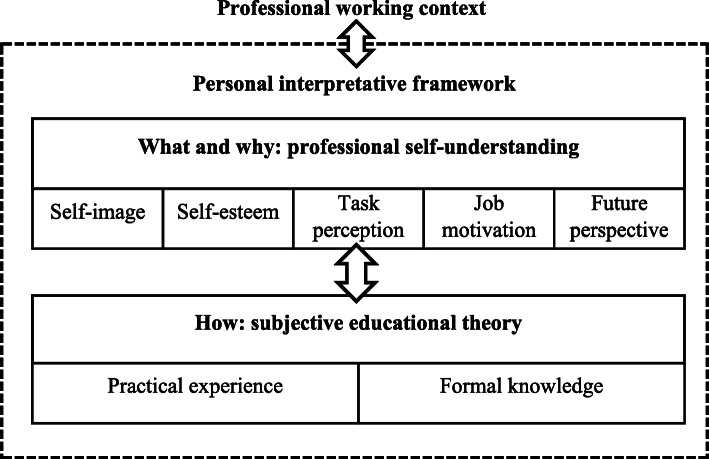
The personal interpretative framework (Kelchtermans 2009). The Personal Interpretative Framework develops from the continuous interaction between mentors and their professional working context. It consists of two dimensions: professional self-understanding and subjective educational theory, which consistently interact, as indicated by the double-headed arrows. Both dimensions consist of multiple components, respectively describing the what, why, and how of mentoring

For the development of the MERIT, we used the *personal interpretative framework* by Kelchtermans [12] to operationalize mentors’ knowledge and beliefs. The personal interpretative framework results from the meaningful interactions between individual mentors and their professional working context. It incorporates two dimensions: *professional self-understanding* and *subjective educational theory* (Fig. 1). These dimensions serve as a lens through which mentors make sense of, and respond to, their practice and experiences. Professional self-understanding refers to how mentors see themselves in their profession. It entails their self-image, self-esteem, task perception, job motivation, and future job perspective, and it can be seen as the mentor’s personal goals and norms (i.e. the ‘*what* I do and *why* I mentor’). Subjective educational theory involves the personal knowledge and beliefs mentors use to decide how to act in specific situations, encompassing the ‘*how* to’ of mentoring. It is based on personal experience, but also, among other things, knowledge from formal training initiatives and observation of other mentors on the job.

The subjective educational theory is an idiosyncratic construct, representing ‘what works’ for specific individuals. This means that one mentor’s framework is not necessarily the indisputable truth for others. Deciding on the most adequate approach in a given situation is based on mentor’s subjective educational theory, drawing on previous experiences: ‘What did I do in similar situations in the past, and how did that work out?’ and on elements of a mentor’s professional self-understanding: ‘What do I need to do in order to be a good mentor?’ It is, in other words, the *operationalization* of the mentor’s professional self-understanding and subjective educational theory [12].

The personal interpretative framework has been studied in a number of occupational groups, that is, beginning and experienced teachers, school leaders, teacher educators [9, 10], and, more recently, also mentors within health professions education [13]. When faculty development programs intend to support mentors in making their personal interpretative framework explicit, it is key to assist them in making the framework explicit through critical reflection [14]. Critical reflection can lead to a significant learning experience because it moves beyond reflection on action [15] in the direction of thinking about what underpins mentors’ practice and critically evaluating the what, how and why of this practice [12, 16, 17].

Previous research suggests that teachers are able to use a survey instrument to explore their teaching conceptions [18, 19]. Although the development of instruments for evaluating professional identity formation [20] or evaluating the development of mentoring processes [3] has been encouraged in the literature, currently no survey instrument is available that supports mentors in making their mentoring knowledge and beliefs explicit [8, 21, 22]. Therefore, the purpose of the current study was to develop and collect initial validity evidence for a self-report survey instrument that mentors can use to make their personal interpretative framework explicit.

## Methods

We developed a survey instrument, pre-tested an initial set of items through cognitive interviews, collected pilot data, and assessed internal structure and reliability of the final survey based on responses from an international sample of mentors.

### Development of the survey

The first version of the survey consisted of 33 agree-disagree items about personal self-understanding (four subscales) and subjective educational theory (five subscales). These subscales were based on previous work with mentors in health professions education [13]. All items were extensively discussed in two rounds by the research team, which consisted of three educational experts (LML: cognitive and educational psychology, EV: educational sciences, EWD: educational sciences and medical education), one cognitive psychologist (PWMVG), and one educational psychologist and medical education researcher specialized in construction and use of surveys (ARA). Discussions on the development of the survey centered on item quality, uniqueness or redundancy, phrasing, and omission of items.

In the second version of the survey, items were formulated in such a way that they aimed at mentors’ and mentees’ goals (seeing the mentee either as future health professional or developing individual), and on whether the mentor-mentee relationship was predominantly mentor or mentee directed. This version contained 24 items using a five-point response scale: not at all true of me, slightly true of me, somewhat true of me, mostly true of me, completely true of me [23]. The revised version was subjected to two rounds of cognitive interviews [24]. During the cognitive interviews, four respondents (a mix of men and women, both junior and more senior mentors working in medicine, education, medical education, and psychology) completed the survey in the presence of LML, followed by a think-aloud protocol interleaved with probe questions, such as: “Can you describe [term] in your own words?” and “Why were you doubting your initial answer?” After the first round of four interviews, six questions were removed because they were unclear, six questions were rewritten because they were deemed ambiguous, and two questions about reasons to mentor were added, resulting in a total of 20 questions. Furthermore, questions were re-ordered, clustered more thematically, and preceded by probes like “As a mentor, my goal is to: …” Also, the overall instruction to “think about how you *actually* mentor, instead of how you think you *should* mentor” was included in the survey information, to make sure that mentors drew from their theories-in-use instead of from their espoused theories [25]. Following these changes, two additional cognitive interviews with respondents from the first round (a clinician and an educationalist) were conducted. At this stage, only minor textual changes to the survey were made. The final online survey was formatted and ultimately administered in Qualtrics (Provo, Utah).

A pilot study with 20 respondents (mentors in health professions education at Maastricht University) was conducted with the 20-item survey. This pilot did not result in further changes to the survey items. Therefore, responses from the pilot were included in the sample. The final survey (see Additional file 1) also included an eight-item demographic section.

### Survey distribution; sample and data collection

For this study, mentors in health professions education were defined as faculty members who had a formal mentoring relationship with one or multiple (under)graduate students. The focus of this relationship was on supporting personal or professional learning and development of the student through supporting competency development and reflection (after Nicholls [11]). We excluded mentors who worked with postgraduate learners, or mentors outside the realm of health professions education.

We distributed the survey through our professional contacts with 137 personal e-mails, three e-mail lists, three Twitter accounts (around 4000 cumulative followers) and LinkedIn. Contact persons were approached with a standardized e-mail, asking them whether they were willing to distribute the survey invitation to mentors in their network. Contact persons who agreed, received a template e-mail that they could forward to their colleagues. The templates contained a link and QR code referring to the online survey. Twitter distribution was done with tweets on the personal accounts of ARA, EWD, and LML. All responses to the survey were collected anonymously. Individual mentors who wished to receive their personal and aggregated sample answers to the survey could provide their e-mail addresses at the end of the survey. Answers were then provided to them based on the connection between their mail address and a randomly generated personal identifier. For each completed survey, €1 was donated to Doctors Without Borders (https://www.doctorswithoutborders.org/).

### Testing psychometric properties of the survey: data analysis

To explore the internal structure of the survey scores, we conducted an exploratory factor analysis (EFA) using Principal Axis Factoring (PAF). Once factors were identified, we calculated the internal consistency reliability of the subscale scores (Cronbach’s alpha) and then created unweighted mean scores for the items that comprised each of the factors. We also calculated descriptive statistics for the total sample. All calculations were done using IBM SPSS statistical software, version 25 (IBM Corporation, New York) and Microsoft Excel 2016 (Microsoft Corporation, Redmond, Washington).

### Ethical approval

This research was approved by the Maastricht University Ethics Review Committee (UM-REC), file number: FHML-REC/2019/033, October 1, 2019.

## Results

### Survey distribution

The 137 e-mails sent to contact persons yielded 50 positive responses (37%), 15 (11%) declines, and 72 (52%) non-responders or undeliverable e-mails. Because the survey was distributed via contact persons and social media, it was not possible to know the overall denominator and, thus, we could not calculate an overall response rate for the survey. However, because this initial study was intended to explore the internal structure of the survey, as opposed to characterize a population, the lack of a response rate is less problematic [26].

To achieve a stable factor structure, we aimed to obtain at least ten responses per survey item as recommended by Pett, Lackey [27] and Stevens [28]. This number was reached in February 2020, after which we kept the survey open until April 1, 2020, resulting in 32 additional responses. After removing four responses from mentors outside health professions education, 228 completed surveys remained and were analyzed.

### Respondent demographics

Seventy-seven (34%) of our mentors identified as men, 148 (65%) as women, one respondent indicated ‘other’ and two respondents (1%) did not identify their gender. The average age of 225 respondents was 46 years (range = 26–72 years) (see Additional file 2, Table 1). Three mentors did not reveal their age. Most mentors (137, 60%) indicated that they mentored individuals in medicine, and that they had an average of 9 years (range = 0–57 years) of mentoring experience (see Additional file 2, Table 2).

**Table 1:** Factor scores and Cronbach’s alphas (α) of MERIT survey items

Factor Name	Survey Item	Percentage explained variance	***α***	Factor scores	Mean score and standard deviation (***SD***) per factor
**Supporting personal development**		19.4%	.75		*M* = 4.3 (*SD* = .55)
	Helping my mentees develop into their own individual person is my reason to mentor.			.810	
	Helping my mentees optimize their wellbeing is my reason to mentor.			.552	
	Helping my mentees become better learners is my reason to mentor.			.306	
	Helping my mentees envision what kind of professional they want to be in the future is my reason to mentor.			.590	
	The personal development of my mentee is extremely important for me as mentor.			.658	
**Modelling professional development**		9.3%	.56		*M* = 3.7 (*SD* = .58)
	I provide my mentees with insights into how the academic world works.			.384	
	I advise my mentees what they should do based on my own experiences			.578	
	If my mentees want feedback on how they are doing, they should ask me for it.			.496	
	I want my mentees to adhere to my professional norms.			.335	
	I am a sort of “help desk” for my students, providing them with information or referring them to resources.			.423	
**Fostering autonomy**		6.6%	.54		*M* = 3.7 (*SD* = *.*71)
	It is my mentees’ own responsibility to ask me for advice if they have any questions			.496	
	I cannot solve problems for my mentees, they have to do that themselves.			.490	
	There is a limit to the amount of support I am prepared to give to my mentees.			.321	
**Monitoring performance**		8.0%	.42		*M* = 3.8 (*SD* = .74)
	I help my mentees gain better understanding of the results of their actions.			.307	
	I am my mentees’ trusted person within the university.			.431	
	Having access to progress indicators of my mentee is critical for me as mentor.			.395	
	If my mentees fail to meet expected performance standards, I will let them know.			.604	

### Testing psychometric properties of the survey

#### Principal Axis factoring

To extract factors from our dataset, we conducted Principal Axis Factoring with direct oblique (Oblimin) rotation (Delta = 0). To be retained in the final solution, factor loadings for individual items had to be greater than 0.3. For the purpose of this analysis, the number of factors to be retained was determined based on several criteria [29], including parallel analysis, examination of the resulting scree plot, and eigenvalues greater than 1.0 (i.e., the K1 criterion). The parallel analysis, which compares mean eigenvalues from randomly generated data to the actual eigenvalues from the mentoring items, suggested four factors to be retained. This four-factor result, however, was neither supported by the K1 criterion, which suggested six initial factors, nor was it supported by an inspection of the scree plot, which also suggested six factors. Based on the results of the parallel analysis, the scree plot and the K1 criterion, it was decided to retain four factors, accounting for 43% of the variance of all items. The four-factor solution was preferred, considering the risk of specifying too many factors, which can lead to many uninformative factors [27].

The four factors are presented in Table 1. Three items had factor loadings less than 0.3: “I can help my mentees to solve problems”, “My relationship with my mentees is based on an equal power balance” and “The amount of support I provide depends on the needs of each of my mentees”. These three items were therefore dropped from further analysis.

The items which clustered in factor one all centered on the personal development of the mentee, hence the factor was named *supporting personal development*. Factor two was indicated as *modelling professional development* and comprised of items that relate to the topic of helping mentees socialize into the academic world and supporting them in picking up scientific norms and values. Factor three, called *fostering autonomy*, primarily represented items about advice-seeking behavior and problem solving. Factor four, *monitoring performance*, addressed understanding and accessing mentees’ performance results and meeting performance standards.

#### Reliability analysis

Cronbach’s alpha of the first factor (modelling professional development) was *α* = .75. The Cronbach’s alpha for the other three factors varied between .42 and .56 (see Table 1) [30]. Deleting items from the factors did not increase their reliability.

#### Item frequencies

On the item level, the average answers ranged from 3.2 to 4.5 on the five-point response scale, with an overall mean of 3.97 (*SD* = 0.89). Thus, on average, mentors indicated that items were at least mostly true or completely true of them (see Additional file 2, Table 3).

## Discussion

The aim of this study was to develop and collect initial validity evidence for the MERIT, an instrument aimed to stimulate reflection in order to make explicit mentors’ personal interpretative framework based on four factors: (1) supporting personal development, (2) modelling professional development, (3) fostering autonomy, and (4) monitoring performance. The scores on the MERIT items were high overall, but varied sufficiently, which demonstrates the value of the instrument for gaining insight into mentors’ knowledge and beliefs. We suggest interpreting the four factors as focus points for how mentors see their own mentoring. Some mentors might focus primarily on mentees’ personal development, others more on professional development, autonomy, performance, or a combination of multiple of these focus points. Mentors can gain insight in which factors are prioritized in their mentoring practice, identify potential gaps or tensions between their theory in use and espoused theory, and decide on actions to close these gaps or reduce tension.

The way the survey items clustered into factors suggests that there is no clear division between professional self-understanding and subjective educational theory. This aligns with the starting premise of the personal interpretative framework: the framework consists of two subdomains which can be analytically distinguished from one another, but are intertwined in practice [12]. This also has practical implications for how mentors can interpret the focus of their mentoring. Reflection on their personal interpretative framework should take a combined approach: they should not only think about what they did and what the subsequent result was, but also consider which beliefs underpin their practice. Combining reflection on action [15] with reflection on knowledge and beliefs of mentoring can lead to a deeper understanding of why and how they mentor. The combination of professional self-understanding and subjective educational theory into one instrument allows the MERIT survey to provide an overview of the how, what, and why of mentoring: which tasks do mentors take on as part of their role, which not, why is this the case, and how do mentors enact their mentoring? Reading the items can also raise awareness about other ways of mentoring because items show that it is possible to mentor in different ways.

The MERIT may not only be used for individual purposes, but also as a precursor for collaborative activities. Making the personal interpretative framework explicit and discussing it with peers can allow others to react on these reflections, question, confirm, or contradict them, and thereby foster the understanding of a mentors’ personal interpretative framework. Discussing the framework with peers serves as an additional stimulus for deep reflection: it invites mentors to think about, and explain why they enact their role in a certain way and it can help them consider alternative approaches to mentoring or points to focus on [12]. The outcomes of these self-reflections can be used in discussions on which approach to mentoring fits best in which situation [31], but also in other faculty development formats [32, 33]. Examples of this type of initiatives are peer supervision, coaching [34, 35], case-based simulations or role-playing critical incidents [3, 36]. These could prove to be far more valuable than discussing instrumental knowledge or trying to convince mentors of a particular approach for mentoring based on theory (e.g., “the literature has shown that x or y is more effective”) [37]. These context-based, reflective sessions can give beginning mentors the safety net that they often seek: There is not one correct way of mentoring, but a wide range of approaches that work in various situations [4, 13].

Our study has a number of important limitations. First, due to the way we distributed the survey, we were unable to calculate a response rate and to check whether respondents were representative for mentors in the field of health professions education. Also, despite our efforts to distribute the survey globally, the vast majority of the respondents fulfilled mentoring roles in Europe (73.3%) and North America (18.9%). Second, the current study did not explicitly consider the possible impact of mentor and mentee characteristics, like gender, ethnicity, or age, on the personal interpretative framework of mentors. In future work, the impact of these characteristics, as well as contextual factors, such as programmatic requirements to mentoring, on the personal interpretative framework can be investigated with an analysis of covariance. Third, the survey in its current configuration showed a substantial variation in reliability (Cronbach’s alpha) across the four factors. Further development of the survey, with regard to both content and internal structure, is therefore warranted. In particular, the adaptation of existing, or the development of additional, items to the subscales with low reliability may be required. Because respondents scored high on most items, questions could be added that require mentors to take a clear position regarding different aspects of the mentoring role (e.g., forced-choice questions), which could lead to a better differentiation of their beliefs. After modification of the item sets, additional data should be collected and confirmatory factor analysis should further validate the factor structure of the instrument. Given both the goal of our study and the context specificity of our theoretical framework, we must interpret our survey results as a first necessary step to explore the internal structure of the MERIT. As such, this effort should not be considered the final step in validating this mentoring survey. From our perspective, the current value of the MERIT lies in helping mentors become aware of their personal interpretative framework and points of focus during their mentoring.

## Conclusion

Administering the MERIT survey in the current international sample of mentors has revealed four factors regarding mentors’ personal interpretative framework: supporting personal development, modelling professional development, fostering autonomy, and monitoring performance. The current version of the MERIT can help mentors gain insight in their knowledge and beliefs about mentoring, based on these four focus points. These insights can serve as valuable feedback for individual mentors and as input for faculty development initiatives, paving the way for mentors’ further professional development.

## Supplementary Information

**Additional file 1.** MERIT survey questions.

**Additional file 2: Table 1.** Personal characteristics of the 228 respondents to the MERIT survey. **Table 2.** Mentoring and mentor setting characteristics of the 228 respondents to the MERIT survey. **Table 3.** Mean, median, mode and SD on item level, frequencies of answers given per MERIT item. List ordered from highest to lowest average.

## Data Availability

The survey instrument used for this study is available as additional digital file 1. The dataset analyzed during the current study is available from the corresponding author on reasonable request.

## References

[1] Skjevik EP, Boudreau JD, Ringberg U, Schei E, Stenfors T, Kvernenes M (2020). Group mentorship for undergraduate medical students—a systematic review. Perspect Med Educ.

[2] Ramani S, Gruppen L, Kachur EK (2006). Twelve tips for developing effective mentors. Med Teach.

[3] Heeneman S, de Grave W (2019). Development and initial validation of a dual-purpose questionnaire capturing mentors’ and mentees’ perceptions and expectations of the mentoring process. BMC Med Educ.

[4] Sambunjak D, Straus SE, Marusić A (2010). A systematic review of qualitative research on the meaning and characteristics of mentoring in academic medicine. J Gen Intern Med.

[5] Sambunjak D, Straus SE, Marusić A (2006). Mentoring in academic medicine: a systematic review. JAMA..

[6] Driessen EW, Overeem K. Mentoring. In: Walsh K, editor. Oxford Textbook of Medical Education. Oxford: Oxford University Press; 2013. p. 265–84.

[7] Driessen EW, Overeem K, van der Vleuten CPM (2011). Get yourself a mentor. Med Educ.

[8] Sng JH, Pei Y, Toh YP, Peh TY, Neo SH, Krishna LKR (2017). Mentoring relationships between senior physicians and junior doctors and/or medical students: a thematic review. Med Teach.

[9] Kelchtermans G (1993). Getting the story, understanding the lives: from career stories to Teachers' professional development. Teach Teach Educ.

[10] Vanassche E, Kelchtermans G (2016). A narrative analysis of a teacher educator’s professional learning journey. Eur J Teach Educ.

[11] Nicholls G, Jarvis P (2006). Mentoring: the art of teaching and learning. The Theory & Practice of teaching.

[12] Kelchtermans G (2009). Who I am in how I teach is the message: self-understanding, vulnerability and reflection. Teachers Teaching.

[13] Loosveld LM, Van Gerven PWM, Vanassche E, Driessen EW (2020). Mentors’ beliefs about their roles in health care education: a qualitative study of mentors’ personal interpretative framework. Acad Med.

[14] Dugdill L, Coffey M, Coufopoulos A, Byrne K, Porcellato L (2009). Developing new community health roles: can reflective learning drive professional practice?. Reflective Pract.

[15] Schön DA (1983). The reflective practitioner: how professionals think in action.

[16] Aspfors J, Fransson G (2015). Research on mentor education for mentors of newly qualified teachers: a qualitative meta-synthesis. Teach Teach Educ.

[17] Pratt DD, Schrewe B, Pusic MV (2019). Pedagogical validity: the key to understanding different forms of ‘good’ teaching. Med Teach.

[18] Jacobs JCG, Wilschut J, van der Vleuten C, Scheele F, Croiset G, Kusurkar RA (2020). An international study on teachers’ conceptions of learning and teaching and corresponding teacher profiles. Med Teach.

[19] Jacobs JCG, Van Luijk SJ, Van Berkel H, Van der Vleuten CP, Croiset G, Scheele F (2012). Development of an instrument (the COLT) to measure conceptions on learning and teaching of teachers, in student-centred medical education. Med Teach.

[20] Tagawa M (2019). Development of a scale to evaluate medical professional identity formation. BMC Med Educ.

[21] Chen Y, Watson R, Hilton A (2016). A review of mentorship measurement tools. Nurse Educ Today.

[22] Berk RA, Berg J, Mortimer R, Walton-Moss B, Yeo TP (2005). Measuring the effectiveness of faculty mentoring relationships. Acad Med.

[23] Artino AR, La Rochelle JS, Dezee KJ, Gehlbach H (2014). Developing questionnaires for educational research: AMEE guide no. 87. Med Teach.

[24] Willis GB, Artino AR (2013). What do our respondents think We're asking? Using cognitive interviewing to improve medical education surveys. J Grad Med Educ.

[25] Argyris C, Schön DA (1974). Theory in practice: increasing professional effectiveness.

[26] Baker R, Brick JM, Bates NA, Battaglia M, Couper MP, Dever JA (2013). Summary report of the AAPOR task force on non-probability sampling. J Survey Stat Methodol.

[27] Pett MA, Lackey NR, Sullivan JJ (2003). Making sense of factor analysis: the use of factor analysis for instrument development in health care research.

[28] Stevens J (2009). Applied multivariate statistics for the social sciences. 5th ed. ed.

[29] Henson R, Roberts J (2006). Use of exploratory factor analysis in published ResearchCommon errors and some comment on improved practice. Educ Psychol Measurement EDUC PSYCHOL MEAS.

[30] McCoach DB, Gable RK, Madura JP (2013). Instrument development of the affective domain. School and corporate applications. Educacion Quimica.

[31] Straus SE, Chatur F, Taylor M (2009). Issues in the Mentor–mentee relationship in academic medicine: a qualitative study. Acad Med.

[32] Steinert Y, Mann K, Anderson B, Barnett BM, Centeno A, Naismith L (2016). A systematic review of faculty development initiatives designed to enhance teaching effectiveness: a 10-year update: BEME guide no. 40. Med Teach.

[33] Steinert Y, Mann K, Centeno A, Dolmans D, Spencer J, Gelula M (2006). A systematic review of faculty development initiatives designed to improve teaching effectiveness in medical education: BEME guide no. 8. Med Teach.

[34] McLeod PJ, Steinert Y (2009). Peer coaching as an approach to faculty development. Med Teach.

[35] O’Keefe M, Lecouteur A, Miller J, McGowan U (2009). The colleague development program: a multidisciplinary program of peer observation partnerships. Med Teach.

[36] Branch WT (2005). Use of critical incident reports in medical education. A perspective. J Gen Intern Med.

[37] Vanassche E, Kelchtermans G (2015). Facilitating self-study of teacher education practices: toward a pedagogy of teacher educator professional development. Prof Dev Educ.

