# Supplementary material for: MERIT: a mentor reflection instrument for identifying the personal interpretative framework
Source: BMC Med Educ. 2021 Mar 4;21:144. doi: 10.1186/s12909-021-02579-x (PMC7934546; doi:10.1186/s12909-021-02579-x)
Supplement: Supplementary file 1 — Additional file 1. MERIT survey questions. [file 12909_2021_2579_MOESM1_ESM.docx]

MERIT: A Mentor Reflection Instrument for Identifying the Personal Interpretative Framework

Lianne M. Loosveld^a^

Pascal W.M. Van Gerven ^a^

Erik W. Driessen ^a^

Eline Vanassche ^b^

Anthony R. Artino Jr. ^c^

**MERIT Survey Questions**

**Answer options** (presented as radio buttons in online survey)**:**

1. Completely untrue for me

2. Somewhat untrue for me

3. Neither true nor untrue for me

4. Somewhat true for me

5. Completely true for me

**Why I mentor**

My reason to mentor is to help my mentees develop into their own individual person.

My reason to mentor is to help my mentees optimize their wellbeing.

My reason to mentor is to help my mentees become better learners.

My reason to mentor is to help my mentees envision what kind of professional they want to be in the future.

**Who I am and what I do as mentor**

As a mentor, I am a sort of "help desk" for my students, providing them with information or referring them to resources.

As a mentor, I provide my mentees with insights into how the academic world works.

As a mentor, I help my mentees gain better understanding of the results of their actions.

As a mentor, I am my mentees' trusted person within the university.

**What is important for me as mentor**

For me as mentor, the personal development of my mentee is extremely important.

For me as mentor, having access to progress indicators of my mentee is critical.

**Advice and problem solving**

It is my mentees' own responsibility to ask me for advice if they have any questions

I advise my mentees what they should do based on my own experiences

I cannot solve problems for my mentees, they have to do that themselves.

I can help my mentees to solve problems

**Feedback**

If my mentees fail to meet expected performance standards, I will let them know.

If my mentees want feedback on how they are doing, they should ask me for it.

**Providing support**

I want my mentees to adhere to my professional norms.

My relationship with my mentees is based on an equal power balance.

The amount of support I provide depends on the needs of each of my mentees.

There is a limit to the amount of support I am prepared to give to my mentees.

**Demographic and general questions:**

- In which educational program do you primarily mentor?
- In which country do you primarily mentor?
- How many years of mentoring experience do you have?
- Do you have to assess your mentee (on any aspect of their functioning)?
- What is your year of birth?
- What is your gender?
- What is your own initial training (multiple answers possible)?
- What do you see as your current 'main profession' (please select one option)?
- You have now answered a number of items on mentoring. These items may or may not have encompassed the full complexity of your daily mentoring practice. Are there any aspects of mentoring that were not (sufficiently) covered in this survey?
- Are there any remarks you would wish to make on this survey (e.g. design, complexity, etc.)?
- Are you interested in receiving your personal answers to this survey?
