# Supplementary material for: MERIT: a mentor reflection instrument for identifying the personal interpretative framework
Source: BMC Med Educ. 2021 Mar 4;21:144. doi: 10.1186/s12909-021-02579-x (PMC7934546; doi:10.1186/s12909-021-02579-x)
Supplement: Supplementary file 2 — Additional file 2: Table 1. Personal characteristics of the 228 respondents to the MERIT survey. Table 2. Mentoring and mentor setting characteristics of the 228 respondents to the MERIT survey. Table 3. Mean, median, mode and SD on item level, frequencies of answers given per MERIT item. List ordered from highest to lowest average. [file 12909_2021_2579_MOESM2_ESM.docx]

MERIT: A Mentor Reflection Instrument for Identifying the Personal Interpretative Framework

Lianne M. Loosveld^a^

Pascal W.M. Van Gerven ^a^

Erik W. Driessen ^a^

Eline Vanassche ^b^

Anthony R. Artino Jr. ^c^

**Table 1** Personal characteristics of the 228 respondents to the MERIT survey.

| **Variable** | **No. of respondents (% of 228)** |
| --- | --- |
| **Gender** |  |
| Women | 148 (65%) |
| Men | 77 (34%) |
| Other | 1 (.5%) |
| Unanswered | 2 (1%) |
| **Age** |  |
| 26 – 35 years | 45 (20%) |
| 36 – 45 years | 72 (32%) |
| 46 – 55 years | 51 (22%) |
| 56 – 65 years | 45 (20%) |
| 66 – 75 years | 12 (5%) |
| Unanswered | 3 (1%) |
| **Initial training of mentor** |  |
| Medicine | 121 (53.1%) |
| Educational Sciences | 41 (18.0%) |
| Health Sciences | 35 (15.4%) |
| Psychology | 24 (10.5%) |
| Biomedical Sciences | 18 (7.9%) |
| Basic Sciences | 13 (5.7%) |
| Social Sciences | 10 (4.4%) |
| Allied Health Professions | 8 (3.5%) |
| Public Health | 6 (2.6%) |
| Nursing Sciences | 2 (0.9%) |
| Pharmacy | 2 (0.9%) |
| Other | 22 (9.6%) |
| **Current main profession** |  |
| Clinician | 81 (35.5%) |
| Researcher | 45 (19.7%) |
| Teacher/Educator | 42 (18.4%) |
| Educationalist | 23 (10.1%) |
| PhD Candidate | 16 (7.0%) |
| Basic Scientist | 5(2.2%) |
| Other | 16 (7.0%) |

**Table 2** Mentoring and mentor setting characteristics of the 228 respondents to the MERIT survey.

| **Variable** | **No. of respondents (% of 228)** |
| --- | --- |
| **Educational Program in which mentor mentors** |  |
| Medicine | 137 (60.1%) |
| Health Sciences | 33 (14.5%) |
| Educational Sciences | 22 (9.6%) |
| Biomedical Sciences | 19 (8.3%) |
| Allied Health Professions | 5 (2.2%) |
| Pharmacy | 2 (0.9%) |
| Public Health | 1 (0.4%) |
| Dentistry | 1 (0.4%) |
| Other | 8 (3.5%) |
| **Country in which mentor mentors (per continent)** |  |
| Europe | 168 (73.3%) |
| North America | 43 (18.9%) |
| Australia | 8 (3.5%) |
| Asia | 6 (2.6%) |
| Africa | 3 (1.3%) |
| **Years of mentoring experience** |  |
| 0-5 | 99 (43.4%) |
| 6-10 | 64 (28.1%) |
| 11-15 | 31 (13.6%) |
| 16-20 | 14 (6.1%) |
| 21-25 | 13 (5.7%) |
| 26-30 | 7 (3.1%) |
| 31-35 | 2 (0.9%) |
| 36-40 | 1 (0.4%) |
| 41-45 | 0 (0.0%) |
| 46-50 | 0 (0.0%) |
| 51-55 | 0 (0.0%) |
| 56-60 | 1 (0.4%) |
| **Mentor assesses mentee** |  |
| Yes | 180 (78.9%) |
| No | 41 (18.0%) |
| Don’t know | 7 (3.1%) |

**Table 3** Mean, median, mode and SD on item level, frequencies of answers given per MERIT item. List ordered from highest to lowest average.

| **ITEM** | **Mean** | **Median** | **Mode** | **Standard Deviation** | **Factor** | **Frequencies per answer value (1 to 5)** | | | | |
| --- | --- | --- | --- | --- | --- | --- | --- | --- | --- | --- |
|  |  |  |  |  |  | **Not at all true of me** | **Slightly true of me** | **Somewhat true of me** | **Mostly true of me** | **Completely true of me** |
| The personal development of my mentee is extremely important for me as mentor. | 4.5 | 5 | 5 | 0.72 | 1 | 1 | 3 | 16 | 74 | 134 |
| The amount of support I provide depends on the needs of each of my mentees. | 4.4 | 5 | 5 | 0.73 | - | 1 | 5 | 12 | 84 | 126 |
| Helping my mentees develop into their own individual person is my reason to mentor. | 4.4 | 5 | 5 | 0.81 | 1 | 2 | 6 | 18 | 79 | 123 |
| Helping my mentees envision what kind of professional they want to be in the future is my reason to mentor. | 4.4 | 4 | 5 | 0.77 | 1 | 1 | 6 | 18 | 91 | 112 |
| If my mentees fail to meet expected performance standards, I will let them know. | 4.2 | 4 | 5 | 0.93 | 4 | 5 | 10 | 22 | 94 | 97 |
| Helping my mentees become better learners is my reason to mentor. | 4.1 | 4 | 4 | 0.77 | 1 | 1 | 5 | 32 | 112 | 78 |
| I can help my mentees to solve problems | 4.1 | 4 | 4 | 0.75 | - | - | 10 | 22 | 126 | 70 |
| Helping my mentees optimize their wellbeing is my reason to mentor. | 4.1 | 4 | 4 | 0.81 | 1 | 2 | 5 | 39 | 109 | 73 |
| I help my mentees gain better understanding of the results of their actions. | 4.1 | 4 | 4 | 0.78 | 4 | 1 | 8 | 32 | 120 | 67 |
| I provide my mentees with insights into how the academic world works. | 4.1 | 4 | 4 | 0.87 | 2 | 3 | 9 | 36 | 106 | 74 |
| It is my mentees' own responsibility to ask me for advice if they have any questions | 4.0 | 4 | 4 | 0.90 | 3 | 1 | 20 | 24 | 114 | 69 |
| I am my mentees' trusted person within the university. | 4.0 | 4 | 4 | 0.91 | 4 | 3 | 12 | 44 | 100 | 69 |
| I am a sort of "help desk" for my students, providing them with information or referring them to resources. | 3.9 | 4 | 4 | 0.92 | 2 | 2 | 20 | 35 | 109 | 62 |
| Having access to progress indicators of my mentee is critical for me as mentor. | 3.9 | 4 | 4 | 1.00 | 4 | 5 | 17 | 50 | 88 | 68 |
| I advise my mentees what they should do based on my own experiences | 3.7 | 4 | 4 | 0.93 | 2 | 2 | 31 | 43 | 116 | 36 |
| There is a limit to the amount of support I am prepared to give to my mentees. | 3.7 | 4 | 4 | 1.12 | 3 | 13 | 30 | 28 | 110 | 47 |
| My relationship with my mentees is based on an equal power balance. | 3.6 | 4 | 4 | 1.01 | - | 3 | 39 | 46 | 101 | 39 |
| I want my mentees to adhere to my professional norms. | 3.6 | 4 | 4 | 0.92 | 2 | 5 | 27 | 58 | 112 | 26 |
| I cannot solve problems for my mentees, they have to do that themselves. | 3.4 | 4 | 4 | 1.12 | 3 | 13 | 38 | 50 | 91 | 36 |
| If my mentees want feedback on how they are doing, they should ask me for it. | 3.2 | 3 | 4 | 1.17 | 2 | 15 | 57 | 55 | 66 | 35 |
| **Overall** | 3.97 | 4.11 | 4.26 | 0.89 |  |  |  |  |  |  |
